# Early mortality and overall survival in oncology phase I trial participants: can we improve patient selection?

**DOI:** 10.1186/1471-2407-11-426

**Published:** 2011-10-05

**Authors:** Nicole G Chau, Ana Florescu, Kelvin K Chan, Lisa Wang, Eric X Chen, Philippe Bedard, Amit M Oza, Lillian L Siu

**Affiliations:** 1 Division of Medical Oncology and Hematology, Princess Margaret Hospital, University of Toronto, Toronto, Canada; 2 Department of Internal Medicine, University of Toronto, Toronto, Canada; 3 Department of Biostatistics; Princess Margaret Hospital, University Health Network, Toronto, Canada

## Abstract

**Background:**

Patient selection for phase I trials (PIT) in oncology is challenging. A typical inclusion criterion for PIT is 'life expectancy > 3 months', however the 90 day mortality (90DM) and overall survival (OS) of patients with advanced solid malignancies are difficult to predict.

**Methods:**

We analyzed 233 patients who were enrolled in PIT at Princess Margaret Hospital. We assessed the relationship between 17 clinical characteristics and 90DM using univariate and multivariate logistic regression analyses to create a risk score (PMHI). We also applied the Royal Marsden Hospital risk score (RMI), which consists of 3 markers (albumin < 35g/L, > 2 metastatic sites, LDH > ULN).

**Results:**

Median age was 57 years (range 21-88). The 90DM rate was 14%; median OS was 320 days. Predictors of 90DM were albumin < 35g/L (OR = 8.2, p = 0.01), > 2 metastatic sites (OR = 2.6, p = 0.02), and ECOG > 0 (OR = 6.3, p = 0.001); all 3 factors constitute the PMHI. To predict 90DM, the PMHI performed better than the RMI (AUC = 0.78 vs 0.69). To predict OS, the RMI performed slightly better (RMI ≥ 2, HR = 2.2, p = 0.002 vs PMHI ≥ 2, HR = 1.6, p = 0.05).

**Conclusions:**

To predict 90DM, the PMHI is helpful. To predict OS, risk models should include ECOG > 0, > 2 metastatic sites, and LDH > ULN. Prospective validation of the PMHI is warranted.

## Background

The primary objectives of phase I trials (PIT) are to define the toxicity profile of a new drug and to determine the dose for further evaluation in phase II trials. Patients enrolled in PIT are therefore placed at risk of toxicity, in exchange for an undefined and limited clinical benefit. Furthermore, patients who are considered for PIT may be regarded as vulnerable because their physical condition may be deteriorating due to advanced malignancy for which no standard treatment options exist.

Selection of appropriate patients for entry onto PIT is therefore critical for patient safety and to achieve study aims. Eligibility criteria are used to minimize risk in study participants and avoid factors that might interfere with determining causality of adverse events. Typical study entry criteria include reasonable performance status, adequate organ functions and an anticipated life expectancy of greater than 3 months. Retrospective reviews by large phase I groups have demonstrated that approximately 25% to 33% of patients do not meet the necessary eligibility criteria at screening [1,2]. In addition, up to 20% of patients die within the first 90 days of PIT entry [3-5]. These early deaths on study are usually attributed to disease progression, as contemporary PIT are safe with a toxic death rate of around 0.5% [3,6-9]. Therefore, the current eligibility process to screen patients may be inadequate, and an objective and reproducible tool is needed to improve patient selection.

To address this need, several single-center, retrospective studies were performed to identify the predictors of survival in patients on PIT [3-6,8-10]. The most recent effort has been the development of a prognostic score to predict survival of patients treated on PIT, [3,4] which was prospectively validated at the Royal Marsden Hospital in the United Kingdom [11]. Derivation of the score was based on a retrospective study of 212 patients treated on PIT at the Royal Marsden Hospital between 2005 to 2006, which revealed in multivariate analysis that the following items were significant independent covariates for poor overall survival (OS): elevated LDH, low albumin and more than 2 sites of metastases [3]. The 3-point prognostic score consisted of normal LDH versus LDH > ULN (score = 0 versus +1), albumin ≥ 35 g/L versus albumin < 35 g/L (score = 0 versus +1), and sites of metastasis ≤ 2 versus > 2 (score = 0 versus +1). In the retrospective study, nearly 90% of patients who died within the first 90 days of study treatment had a risk score of 2 or 3. The authors prospectively validated the prognostic score at the same institution in 78 patients treated in 19 PIT from March 2007 to June 2007 [11]. A significant difference in median survival was again observed for patients in the good-prognosis group (risk score 0 to 1, 33 weeks; 95% CI 24-42 weeks) compared to the poor-prognosis group (risk score 2 to 3, 15.7 weeks; 95% CI 11-21 weeks), and thus the prognostic score was an independent marker for OS (HR = 1.4; p = 0.037; 95% CI 1.02-1.9). The Royal Marsden Hospital Index (RMI) has been validated at other centers in Europe, [12] however the utility of the RMI in North America is unknown.

We performed a retrospective analysis of patients enrolled in PIT at the Princess Margaret Hospital (PMH) over a period of 3.5 years. The goals of the study were: (1) to identify predictors of 90DM in our cohort; (2) to identify predictors of OS; (3) to validate the RMI in a retrospective cohort of patients treated in our institution; and (4) to compare the performance of RMI to that of our index (PMHI) in our cohort.

## Methods

Patients were selected from the PMH Phase I Trials Database, a registry of all patients enrolled on solid tumor PIT at PMH, and included 271 consecutively treated patients who had received at least one dose of treatment, from 1 January 2006 to 1 July 2009. Patient characteristics and vital status were obtained from the PMH Phase I Trials Database, individual patient charts, the PMH Cancer Registry and the Ontario Cancer Registry following approval from the University Health Network Research Ethics Board, the PMH Cancer Registry Database Access Committee and Cancer Care Ontario.

### Statistical Methods

Descriptive statistics were used to summarize the study cohort. All baseline characteristics were examined in univariate analysis as predictors for 90DM and OS using logistic regression and Cox proportional hazards model respectively. Only those which were significant at the 0.10 (two-sided) level in the univariate analysis were entered into the exploratory multivariate analysis, and variables that remained significant at 0.05 (two-sided) level in the multivariate analysis were considered significant prognostic factors. For each individual, the RMI score was also derived from the sum of the three components used in the prognostic model established by the Royal Marsden Hospital [3]. Patients were subcategorized into two groups for the RMI: total score of 0 to 1 (good risk group), and 2 to 3 (poor risk group). The Kaplan-Meier method was used to estimate OS and the log-rank test was used to compare survival curves. The receiver operating characteristic curve was used to measure the discrimination of 90DM by different prognostic indices. The overall concordance index (*C *index) was used to measure the discrimination ability for the survival analysis models generated by the PMHI and RMI, with a value of 0.5 having no discriminative ability and a score of 1.0 having perfect discriminative ability [13]. Statistical analyses were performed using the SAS 9.2 program (SAS Institute, Cary, NC).

## Results

### Baseline patient characteristics

For the analysis of 90DM, we excluded 38 patients (11%) from the cohort of 271 patients enrolled on PIT for the following reasons: primary diagnosis of hematological malignancies (n = 22) and missing data (90DM (n = 5), performance status (n = 10), number of metastatic sites (n = 1)). The baseline patient characteristics of the remaining 233 patients are presented in Table 1. Median age was 57 years (range: 21-88); 53% were male; the majority had an Eastern Cooperative Oncology Group (ECOG) performance status of 1 (52%); and the median number of previous treatments was 2 (range: 0-14). A variety of cancers were represented; the most common was gastrointestinal origin (46%). Overall, 59% had 1 to 2 sites of metastases (median 3, range: 1-4 sites). The most common sites of metastasis were the lung (55%), liver (52%) and bone (17%). The majority of patients were treated with a combination of molecularly targeted and cytotoxic chemotherapeutic agents (58%); 38% were treated with molecularly targeted agents alone. Baseline biochemistry revealed a low albumin (albumin < 35 g/L) in 3% of patients, and elevated LDH (LDH > ULN) in 39% of patients. In terms of blood counts based on conventional laboratory thresholds, hemoglobin of less than 12 g/dL, white blood count of more than 10.5 × 10^9^/L and platelet count of more than 400 × 10^9^/L were present in 39%, 13% and 12% of patients, respectively.

**Table 1:** Baseline patient characteristics, n = 233

Characteristic		Number	%	Median (range)
**Gender**	Male	124	53	

**Age**	Median (range)	57 (21-88)		

**Performance status**	ECOG 0	105	45	

	ECOG 1	122	52	

	ECOG 2	6	3	

**No. of prior treatments**				2 (0-14)

	0-2	135	58	

	≥ 3	98	42	

**Primary tumor site**	Gastrointestinal	108	46	

	Thoracic, Head & Neck	58	25	

	Breast & Gynecological	40	17	

	Urologic: Sarcoma: Other	9: 5: 13	4:2:6	

**Type of trial**	Targeted agent alone	89	38.2	
	Cytotoxic agent alone	8	3.4	
	Combined	136	58.4	

**No. of metastatic sites**				3 (1-4)

	Locoregional disease*	20	9	

	1-2 sites	138	59	

	> 2 sites	75	32	

**Site of metastasis**	Liver	121	52	

	Lung	129	55	

	Bone	39	17	

**Baseline albumin**				41 g/L (28-48 g/L)

	< 35 g/L	8	3	
	≥ 35 g/L	225	97	

**Baseline LDH**				224 U/L (93-3132 U/L)

	Normal LDH	142	61	

	Elevated LDH > ULN	91	39	

**Baseline hemoglobin**				12.5 g/dL (8.2-17.1 g/dL)

	< 12 g/dL	90	39	
	≥ 12 g/dL	143	61	

**Baseline WBC**				7 × 10^9^/L (2.3-20.7 × 10^9^/L)

	≤ 10.5 × 10^9^/L	203	87	
	> 10.5 × 10^9^/L	30	13	

**Baseline platelets**				264 × 10^9^/L (106-832 × 10^9^/L)

	≤ 400 × 10^9^/L	204	88	
	> 400 × 10^9^/L	29	12	

*Patients with locoregional disease were included as ≤ 2 metastatic sites in the analysis

Abbreviations: No. = number; LDH = lactate dehydrogenase; WBC = white blood cells; ULN = upper limit of normal

### Patient outcomes: Early mortality, overall survival, and response

Patient outcomes are described in Table 2. The 90DM in our cohort was 14%. The median survival was 320 days (range: 270-365 days) with a median follow-up time of 224 days (range: 23-1345 days). There were no reported toxic deaths. Of the 233 patients, 196 (84%) were evaluable for objective response by Response Evaluation Criteria in Solid Tumors (RECIST) [14]. The best overall response by RECIST was partial response (PR) in 17 patients (9%), stable disease (SD) in 92 patients (47%), and progressive disease (PD) in 87 patients (44%). The clinical benefit rate (PR + SD) was 56% (109 patients). Of those who achieved SD, the majority had a SD duration of 3-6 months (54%) and about one quarter had SD for over 6 months duration (23%).

**Table 2:** 90 day mortality, overall survival and response data of entire cohort

Outcome		Number	%	Median (days)	95% CI (days)
**90 day mortality**	Alive ≥ 90 days	200	86		
	Alive < 90 days	33	14		
**Overall survival**				320	270-365
**Response by RECIST**	Partial Response	17/196	9		
	Stable Disease	92/196	47		
	Progressive Disease	87/196	44		
**Stable disease duration**	0-3 months	20/86	23		
	3-6 months	46/86	54		
	> 6 months	20/86	23		
**Stable disease duration**	1-2 cycles	13/90	15		
	3-4 cycles	23/90	26		
	5-6 cycles	29/90	32		
	> 6 cycles	25/90	27		

Abbreviations: RECIST = Response Evaluation Criteria In Solid Tumors; CI = confidence interval

### Baseline predictors of 90-day mortality

The results of the univariate and multivariate analyses to investigate predictors of 90DM are shown in Table 3. In multivariate analysis, we found that albumin < 35g/L (p = 0.008), > 2 metastatic sites (p = 0.02) and ECOG > 0 (p = 0.001) were significantly associated with 90DM in our cohort. Using these 3 variables, a risk score was constructed and evaluated (PMHI). One point was assigned to each of the 3 variables. For PMHI score of 0-1, the 90DM rate was 7%, whereas a score of 2-3 was associated with a 90DM rate of 37%.

**Table 3:** Univariate and multivariate predictors of 90 day mortality

	Univariate model			Multivariable model (stepwise)		
	**Odds Ratios**	**95% CI**	**p-value**	**Odds Ratios**	**95% CI**	**p-value**

**Male**	1.42	0.67-3.01	0.36			
**Age ≥ 65 years old**	0.54	0.21-1.38	0.21			
**Albumin < 35 g/L**	11.73	2.66-51.78	0.01	8.24	1.75-38.81	0.008
**LDH > ULN**	1.81	0.86-3.79	0.12			
**Metastatic sites > 2**	3.01	1.42-6.38	0.01	2.57	1.15-5.72	0.02
**ECOG Performance Status > 0**	7.40	2.51-21.81	0.01	6.23	2.06-18.83	0.001
**Hemoglobin < 12 g/dL**	2.47	1.167-5.21	0.02			
**WBC > 10.5 × 10**^**9**^**/L**	2.59	1.04-6.44	0.04			
**Platelets > 400 × 10**^**9**^**/L**	0.97	0.31-2.98	0.95			
**Lymphocytes < 0.7 × 10**^**9**^**/L**	2.21	0.94-5.23	0.07			
**Lung metastases**	2.04	0.92-4.51	0.08			
**Liver metastases**	2.04	0.94-4.43	0.07			
**Bone metastases**	1.41	0.57-3.53	0.46			
**Brain metastases**	6.22	0.38-101.94	0.22			
**Lymph node metastases**	2.16	1.02-4.54	0.04			
**Peritoneal metastases**	0.76	0.28-2.10	0.60			
**Prior treatments ≥ 3**	0.76	0.35-1.64	0.48			

Abbreviations: LDH = lactate dehydrogenase; ULN = upper limit of normal; ECOG = Eastern Cooperative Oncology Group; WBC = white blood cells; CI = confidence interval

We then evaluated the RMI developed by Arkenau *et al. *[11], which consisted of albumin < 35 g/L, > 2 metastatic sites and elevated LDH. When applied to our cohort, univariate and multivariate analyses demonstrated that albumin and > 2 metastatic sites were significant predictors of 90DM; albumin < 35 g/L (OR 8.13 95% CI 1.72-38.37, p = 0.008) and > 2 metastatic sites (OR 2.32, 95% CI 1.02-5.28, p = 0.05). However, LDH was not a significant predictor of 90DM in our cohort (OR 1.58, 95% CI 0.69-3.60, p = 0.28). When RMI was applied to our cohort, the 90DM rates were 11% for a score of 0-1 and 29% for a score of 2-3, respectively.

**Figure 1:**
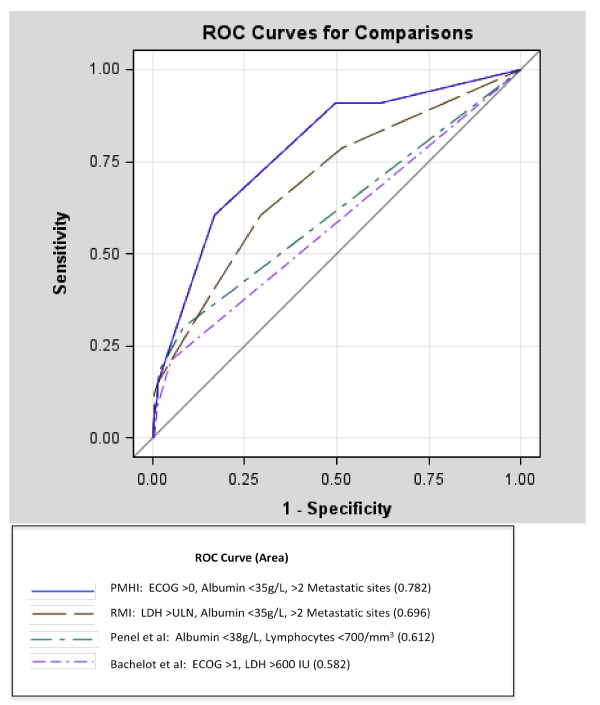
**Receiver operator curve for comparison of PMHI, RMI, Penel index and Bachelot index for predicting 90 day mortality**.

Finally, we compared the test characteristics of the PMHI and RMI in predicting 90DM using a dichotomized score (0-1 vs 2-3) (Table 4). A PMHI score of 2-3 had an OR of 7.24 (95% CI 2.91-18.02), p < 0.0001. A RMI score of 2-3 had an OR of 1.08 (95% CI 0.41-2.89), p = 0.88). The sensitivity of the PMHI for predicting 90DM was 61% with a specificity of 83%. The sensitivity of the RMI for predicting 90DM was 36% with a specificity of 85%. The area under the curve was significantly greater for the PMHI (0.782) compared with the RMI (0.696, 95% CI 0.597-0.794, p = 0.02), or with other indices such as those published by Penel *et al. *[9] (0.612, 95% CI 0.515-0.709, p = 0.001) and Bachelot *et al. *[6] (0.582, 95% CI 0.509-0.654, p = 0.0002) (Figure 1).

**Table 4:** Comparison of test characteristics for predicting 90 day mortality: PMHI vs. RMI using a dichotomized score (0-1 vs 2-3)

Test Characteristic	PMHI	RMI
**Score ≥ 2 in multivariate analysis**	OR 7.24 (95% CI: 2.91-18.02), p < 0.0001	OR 1.08 (95% CI: 0.41-2.89), p = 0.88
**Sensitivity**	61%	36%
**Specificity**	83%	85%
**Positive likelihood ratio**	3.57	2.42
**False positive rate**	14.6%	12.9%
**False negative rate**	5.6%	9.0%
**Area under curve (AUC)**	0.782	0.696

Abbreviations: PMHI = Princess Margaret Hospital Index; RMI = Royal Marsden Hospital Index; OR = odds ratio

### Baseline predictors of overall survival

The results of the univariate and multivariate analyses to investigate predictors of OS are shown in Table 5. In multivariate analysis, we found that high LDH (p = 0.001), > 2 metastatic sites (p = 0.004) and ECOG > 0 (p = 0.05) were significantly associated with OS in our cohort. When the PMHI was applied to predict OS, number of metastatic sites was the only significant predictor (Table 6). By contrast, when using the RMI, both LDH and number of metastatic sites were significant predictors of OS (Table 6). The *C *index for the PMHI is 0.89 (95% CI 0.76-0.98). The *C *index for the RMI is 0.87 (95% CI 0.73-0.96). The median OS according to PMHI and RMI risk score is presented in Table 7.

**Table 5:** Univariate and multivariate predictors of *overall survival*

		Univariate model			Multivariable model (stepwise)		
**Variables**		**Median OS (days)**	**95% CI**	**p-value (Log-rank)**	**Hazard ratio**	**95% CI**	**p-value**

**Gender**	Female	365	276-501	0.05			
	
	Male	270	204-346				

**Age**	< 65 years	325	271-400	0.81			
	
	≥ 65 years	297	210-398				

**Albumin**	≥ 35 g/L	325	271-365	0.02			
	
	< 35 g/L	54	42-408				

**LDH**	Normal	366	320-501	0.001			
	
	Elevated	210	147-276		1.75	1.24-2.46	0.001

**No. of metastatic sites**	≤ 2	358	299-446	0.0001			
	
	> 2	210	136-290		1.68	1.18-2.40	0.004

**ECOG Performance Status**	0	366	302-464	0.02			
	
	> 0	270	158-325		1.42	1.00-1.99	0.05

**Hemoglobin**	≥ 12 g/dL	350	273-412	0.09			
	
	< 12 g/dL	276	185-346				

**WBC**	≤ 10.5 × 10^9^/L	345	276-398	0.07			
	
	> 10.5 × 10^9^/L	236	103-352				

**Platelets**	≤ 400 × 10^9^/L	329	273-398	0.23			
	
	> 400 × 10^9^/L	261	137-352				

**Lymphocytes**	≥ 0.7 × 10^9^/L	346	290-400	0.04			
	
	< 0.7 × 10^9^/L	166	99-315				

**Lung metastases**	No	338	271-414	0.58			
	
	Yes	302	223-365				

**Liver metastases**	No	366	297-501	0.01			
	
	Yes	250	191-329				

**Bone metastases**	No	329	276-365	0.32			
	
	Yes	261	135-433				

**Brain**	No	320	270-358	0.47			
	
	Yes	269.5	40-499				

**Peritoneal Mets**	No	325	250-365	0.99			
	
	Yes	315	257-474				

**No. of prior treatments**	< 3	302	266-365	0.76			
	
	≥ 3	320	227-400				

Abbreviations: LDH = lactate dehydrogenase; No. = number; ECOG = Eastern Cooperative Oncology Group; WBC = white blood cells; OS = overall survival; CI = confidence interval

**Table 6:** Variables associated with *overall survival *in multivariate regression analyses: PMHI vs. RMI

Overall Survival Predictors	Hazard Ratio	95% CI	p-value
**PMHI**			

**Albumin < 35 g/L**	1.99	0.86-4.58	0.11
**> 2 metastatic sites**	1.79	1.26-2.53	0.01
**ECOG Performance Status > 0**	1.36	0.96-1.91	0.08

**RMI**			

**Albumin < 35 g/L**	1.94	0.84-4.48	0.12
**> 2 metastatic sites**	1.74	1.23-2.48	0.01
**LDH > ULN**	1.69	1.20-2.38	0.01

Abbreviations: PMHI = Princess Margaret Hospital Index; RMI = Royal Marsden Hospital Index; ECOG = Eastern Cooperative Oncology Group; LDH = lactate dehydrogenase; ULN = upper limit of normal; CI = confidence interval

**Table 7:** Overall survival according to risk score: Comparison of PMHI and RMI in predicting overall survival

Risk Score	PMHI				RMI			
	No.	Dead	Censored	Median OS (95% CI) in days	No.	Dead	Censored	Median OS (95% CI) in days
**Score 0**	79	41	38	412 (329-515)	104	55	49	400 (325-561)
**Score 1**	100	58	42	320 (239-365)	87	53	34	315 (205-366)
**Score 2**	51	40	11	154 (87-270)	39	31	8	137 (104-223)
**Score 3**	3	3	0	54 (54-408)*	3	3	0	54 (54-408)*

*too few patients in this stratum to provide a stable 95% CI.

Abbreviations: PMHI = Princess Margaret Hospital Index; RMI = Royal Marsden Hospital Index; No. = number; OS = overall survival; CI = confidence interval

**Figure 2:**
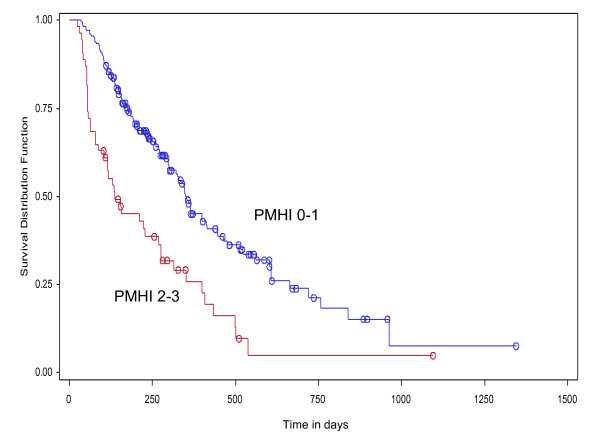
**Kaplan-Meier plot of overall survival by PMHI score 0-1 vs 2-3 (HR 1.58, 95% CI 1.01-2.47, p = 0.05) (log-rank test p < 0.0001)**.

**Figure 3:**
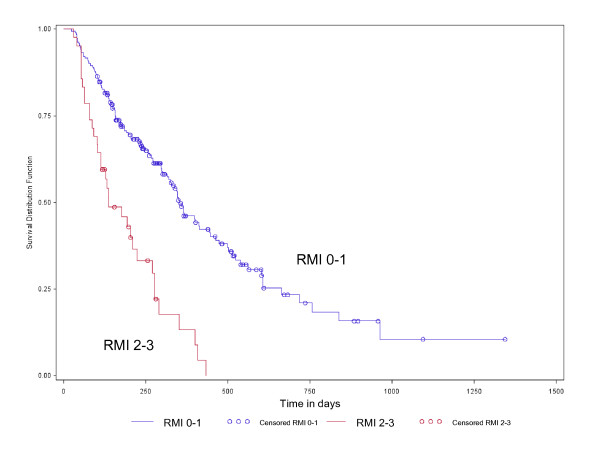
**Kaplan-Meier plot of overall survival by RMI score 0-1 vs 2-3 (HR 2.18, 95% CI 1.33-3.58, p = 0.002) (log-rank test p < 0.0001)**.

Overall, the RMI performed slightly better than the PMHI in predicting OS. A RMI score of 2-3 had a HR of 2.18 (95% CI 1.33-3.58, p = 0.002), while a PMHI score of 2-3 had a HR of 1.58 (95% CI 1.01-2.47, p = 0.05). The Kaplan-Meier survival plot by PMHI score category (0-1 vs 2-3) is illustrated in Figure 2. Patients with PMHI score of 0-1 had a better OS (median OS = 250 days, 95% CI 299-414 days) compared to those with PMHI scores of 2-3 (median OS = 237 days, 95% CI 87-270 days) (log-rank test, p < 0.0001). The survival plot by RMI score category (0-1 vs 2-3) is illustrated in Figure 3. Patients with RMI score of 0-1 had a better OS (median OS = 353 days, 95% CI 315-445 days) than those with RMI scores of 2-3 (median OS = 137 days, 95% CI 104-223 days) (log-rank test p < 0.0001).

### Overall survival is correlated with tumor response

OS was associated with best response by RECIST. Patients with PR had a median OS of 603 days (95% CI 433-756), patients with SD had a median OS of 501 days (95% CI 365-838), and patients with PD had a median OS of 158 days (95% CI 131-205) (log-rank p < 0.0001). Patients with a longer duration of SD had a significantly longer OS. Patients with SD for more than 6 months had a median OS of 561 days (95% CI 412-not reached), patients with SD for 3-6 months had a median OS of 414 days (95% CI 346-not reached) and patients with SD for less than 3 months had a median OS of 185 days (95% CI 135-538) (log-rank p < 0.01). Patients with SD for 3 cycles or more had improved OS (median OS = 538 days, 95% CI 400-719) compared to patients with PD or SD of 1-2 cycles (median OS = 171 days, 95% CI 136-204) (log-rank, p < 0.0001). In addition, OS appeared to be similar for patients with SD for 1-2 cycles (median OS = 171 days, 95% CI 78-not reached) and patients with PD (median OS = 158 days, 95% CI 131-205) (log-rank, p = 0.25).

## Discussion

Selection of patients in oncology PIT remains controversial with often unclear benefit at the cost of potential patient risk [15-17]. Consistent with other studies, [3,4,6-8,11,18] we found a 90-day mortality rate of 14%, while treatment-related deaths were extremely rare (0% in our study). This suggests that early mortality is largely due to cancer progression and it has been proposed that the current patient selection process may be improved with the use of objective selection criteria. We showed that albumin, number of metastatic sites and performance status are independent predictors of 90DM.

The PMHI was superior in predicting 90DM in our patient population, when compared to other risk indices designed to predict mortality in oncology PIT patients [3-6,9]. One explanation may be that a model generated from a single institution's data set will likely perform best in the same institution, compared to an alternative model generated from an external data set. Secondly, the PMHI is derived from predictors of 90DM, which is a clinically relevant and distinct endpoint compared to OS. Whereas, the RMI, which was derived from predictors of OS was superior in predicting OS in our cohort. Thirdly, other risk indices designed to predict 90DM are based on heterogeneous patient populations, including those treated over 10 years ago. Bachelot *et al. *[6] evaluated 154 patients enrolled in phase I trials from 1986 to 1993 and identified two independent risk factors for 90DM (LDH > 600 IU and ECOG > 1). Penel *et al. *[9] analyzed 148 patients who were screened for phase I trial entry from 1997 to 2002 and identified albumin < 38 g/L and lymphocyte count < 700/mm^3 ^to be independent predictors of 90DM. The same authors validated these predictors in a cohort of 128 patients treated with cytotoxic agents from 1986 to 1993 at a separate center [5]. To date, no risk index has been prospectively validated to predict 90DM.

In predicting OS, the PMHI performed well but was slightly inferior to the RMI. The PMHI and RMI are similar in that they both include albumin < 35 g/L and number of metastatic sites. Compared to the RMI, the PMHI substitutes ECOG > 0 for LDH. Our findings may suggest that ECOG is an important predictor of early death within 3 months, whereas LDH, which reflects overall tumor burden, may be a better predictor of total disease burden and OS. The high proportion of patients with a good performance status in our study (ECOG 0 = 45%) compared to the initial study by Arkenau *et al. *[3] (ECOG 0 = 28%) may have contributed to the importance of ECOG in our cohort. In predicting OS in our cohort, both risk indices were likely weakened by the inclusion of albumin < 35 g/L, which was not a significant predictor in multivariate analysis of OS. Albumin < 35 g/L, a marker of nutritional status, was present in only 3% of patients in our cohort, and therefore likely too rare to demonstrate an association with survival.

In keeping with other studies, [6,8,9,11,12,19] we confirmed that other baseline characteristics such as tumor type, number of prior therapies and older age, were not significant predictors of early mortality or OS. This consistent finding affirms that patients should not be excluded from enrolment in oncology PIT on the basis of age, multiple lines of prior therapy or primary tumor histology alone.

Another interesting finding in our analysis is that patients with prolonged SD > 3 cycles had an OS similar to that of patients with PR, and a much better OS than patients with SD of 1-2 cycles and PD. This supports the notion of potential therapeutic benefit with prolonged disease control on contemporary PITs. Indeed, disease control (CR, PR and SD) has been associated with increased survival in patients on PIT [8]. Disease control may be particularly important when evaluating new molecularly targeted agents that may exert greater cytostatic rather than cytotoxic effects. Our study cohort reflects a contemporary PIT patient population treated on modern and often complex phase I protocols. Over 95% of our patients were treated with novel molecularly targeted agents alone or in combination with cytotoxic agents. The median survival in our cohort of 10.7 months is longer than that previously published by several other PIT groups (5.7-9.0 months) [8-11]. The clinical benefit rate in our cohort of 56% is consistent with that reported by other recent studies (45-55%) [3,7,8,20].

There are inherent limitations in this type of analysis. Firstly, the analysis was conducted retrospectively. Although the PMHI was able to identify patients with a greater risk of early mortality in a retrospective cohort, we recognize that our results need to be validated prospectively. Ideally, prospective evaluation of the PMHI should also occur in patients who are being screened for PITs in order to assist with patient decision-making prior to enrolment. Secondly, the study was performed at a single center in North America and therefore the PMHI should be validated across different centers, including those outside of North America. Furthermore, we evaluated patients enrolled in 30 different phase I trials. Although patient heterogeneity may be a confounder in our analysis, it is important to note that tumor histology was not a significant predictor of early mortality in patients enrolled on PIT in our study and previous studies [4,6,9]. Lastly, the findings of PMHI on 90DM should be viewed as exploratory. Given the relatively few number of events of 90DM to the number of predictors, potential bias and overfitting of data exist. A recent simulation study suggests that the commonly used 10:1 rule for the number of predictors per events may be overly stringent and can be relaxed [21]. Therefore, our findings will require careful internal and external validation to confirm validity. Nevertheless, our study cohort represents the largest cohort of PIT patients in North America analyzed for predictors of 90DM.

The PMHI and RMI are useful tools that can assist with patient selection for oncology P1T. In our cohort of unselected patients there were 33 deaths within 90 days of study entry (14%). Limiting patient enrolment to only those with PMHI score 0-1, would result in a reduction of 90DM from 14% to 5.6%. If enrolment was limited to patients with RMI score 0-1, the 90DM would be 9%. However, neither the PMHI or RMI alone is adequate to exclude patients from PIT entry due to the low positive predictive value which would restrict access for some patients who may have benefited. By excluding patients with a PMHI score of 2-3, we would wrongly exclude 34 patients (15%) from study entry, but prevent 20 early deaths on study. Whereas, by excluding patients with a RMI score of 2-3, we would wrongly exclude 30 patients (13%), but prevent 12 early deaths on study.

## Conclusions

Our findings demonstrate that a simple objective risk score consisting of albumin, number of metastatic sites and performance status, can be useful to assist in identifying patients enrolled on PIT who are at a greater risk of early death. This risk score needs to be validated prospectively in other centers.

## Competing interests

The authors declare that they have no competing interests.

## Authors' contributions

NC participated in the study design, data collection, data analysis, drafted and revised the manuscript. AF participated in the data collection, data analysis, drafted and revised the manuscript. KC and LW performed the statistical analysis and revised the manuscript. EC, PB and AO participated in the management of study subjects, reviewed and revised the manuscript. LS participated in the study design and management of study subjects, coordinated the study, performed the data analysis, and drafted and revised the manuscript. All authors read and approved the final manuscript.

## Pre-publication history

The pre-publication history for this paper can be accessed here:

http://www.biomedcentral.com/1471-2407/11/426/prepub

## References

[B1] Ho J Pond G Newman C Maclean M Chen E Oza A Siu L Barriers in phase I cancer clinical trials referrals and enrollment: five-year experience at the Princess Margaret Hospital BMC Cancer 2006 6 263 10.1186/1471-2407-6-263 17092349 PMC1636658

[B2] Karavasilis V Digue L Arkenau T Eaton D Stapleton S de Bono J Judson I Kaye S Identification of factors limiting patient recruitment into phase I trials: a study from the Royal Marsden Hospital Eur J Cancer 2008 44 978 982 10.1016/j.ejca.2008.02.040 18362066

[B3] Arkenau HT Olmos D Ang JE de Bono J Judson I Kaye S Clinical outcome and prognostic factors for patients treated within the context of a phase I study: the Royal Marsden Hospital experience Br J Cancer 2008 98 1029 1033 10.1038/sj.bjc.6604218 18349817 PMC2275488

[B4] Arkenau HT Olmos D Ang JE Burriuso J Karavasilis V Ashley S de Bono J Judson I Kaye S 90-Days mortality rate in patients treated within the context of a phase-I trial: how should we identify patients who should not go on trial? Eur J Cancer 2008 44 1536 1540 10.1016/j.ejca.2008.04.017 18550361

[B5] Penel N Delord JP Bonneterre ME Bachelot T Ray-Coquard I Blay JY Pascal LB Borel C Filleron T Adenis T Bonneterre J Development and validation of a model that predicts early death among cancer patients participating in phase I clinical trials investigating cytotoxics Invest New Drugs 2009 28 76 82 19205623 10.1007/s10637-009-9224-x

[B6] Bachelot T Ray-Coquard I Catimel G Ardiet C Guastalia JP Dumortier A Chauvin F Droz JP Philip T Clavel M Multivariable analysis of prognostic factors for toxicity and survival for patients enrolled in phase I clinical trials Ann Oncol 2000 11 151 156 10.1023/A:1008368319526 10761748

[B7] Horstmann E McCabe MS Grochow L Yamamoto S Rubinstein L Budd T Shoemaker D Emanuel EJ Grady C Risks and benefits of phase I oncology trials, 1991 through 2002 N Engl J Med 2005 352 895 904 10.1056/NEJMsa042220 15745980

[B8] Italiano A Massard C Bahleda R Vataire AL Deutsch E Magne N Pignon JP Vassal G Amand JP Soria JC Treatment outcome and survival in participants of phase I oncology trials carried out from 2003 to 2006 at Institut Gustave Roussy Ann Oncol 2008 19 787 792 18042834 10.1093/annonc/mdm548

[B9] Penel N Vanseymortier M Bonneterre ME Clisant S Dansin E Vendel Y Beuscart R Bonneterre J Prognostic factors among cancer patients with good performance status screened for phase I trials Invest New Drugs 2008 26 53 58 10.1007/s10637-007-9088-x 17891337

[B10] Wheler J Tsimberidou AM Hong D Naing A Jackson T Liu S Feng L Kurzrock R Survival of patients in a Phase 1 Clinic: the M. D. Anderson Cancer Center experience Cancer 2009 115 1091 1099 10.1002/cncr.24018 19165805

[B11] Arkenau HT Barriuso J Olmos D Ang JE de Bono J Judson I Kaye S Prospective validation of a prognostic score to improve patient selection for oncology phase I trials J Clin Oncol 2009 27 2692 2696 10.1200/JCO.2008.19.5081 19332724

[B12] Olmos D Hern RA Marsoni S Tabernero J Soria JC Verweij J Voest E Schoffski P Penel N Schellens J Brunetto A Gianni L Evans J Wilson R Sessa C Plummer R Kaye S Multi-institutional prognostic factor analysis of patients (pts) enrolled in phase I (Ph I) oncology trials: Can pts selection be improved [abstract]? J Clin Oncol (Meeting Abstracts) 2010 28 s2518

[B13] Pencina MJ D'Agostino RB Overall C as a measure of discrimination in survival analysis: model specific population value and confidence interval estimation Stat Med 2004 23 2109 2123 10.1002/sim.1802 15211606

[B14] Therasse P Arbuck SG Eisenhauer EA Wanders J Kaplan RS Rubinstein L Verweij J Van Glabbeke M van Oosterom AT Christian MC Gwyther SG New guidelines to evaluate the response to treatment in solid tumors J Natl Cancer Inst 2000 92 European Organization for Research and Treatment of Cancer, National Cancer Institute of the United States, National Cancer Institute of Canada 205 216 10.1093/jnci/92.3.205 10655437

[B15] Joffe S Miller FG Rethinking risk-benefit assessment for phase I cancer trials J Clin Oncol 2006 24 2987 2990 10.1200/JCO.2005.04.9296 16809725

[B16] Markman M Further evidence of clinical benefit associated with participation in phase I oncology trials Br J Cancer 2008 98 1021 1022 10.1038/sj.bjc.6604206 18349816 PMC2275490

[B17] Seidenfeld J Horstmann E Emanuel EJ Grady C Participants in Phase 1 Oncology Research Trials: Are They Vulnerable? Arch Intern Med 2008 168 16 20 10.1001/archinternmed.2007.6 18195190

[B18] Roberts TG Jr Goulart BH Squitieri L Stallings SC Halpern EF Chabner BA Gazelle GS Finkelstein SN Clark JW Trends in the risks and benefits to patients with cancer participating in phase 1 clinical trials JAMA 2004 292 2130 2140 10.1001/jama.292.17.2130 15523074

[B19] Han C Braybrooke JP Deplanque G Taylor M Mackintosh D Kaur K Samouri K Ganesan TS Harris AL Talbot DC Comparison of prognostic factors in patients in phase I trials of cytotoxic drugs vs new noncytotoxic agents Br J Cancer 2003 89 1166 1171 10.1038/sj.bjc.6601218 14520440 PMC2394292

[B20] Postel-Vinay S Arkenau HT Olmos D Ang J Barriuso J Ashley S Banerji U de Bono J Judson I Kaye S Clinical benefit in Phase-I trials of novel molecularly targeted agents: does dose matter? Br J Cancer 2009 100 1373 1378 10.1038/sj.bjc.6605030 19401696 PMC2694416

[B21] Vittinghoff E McCulloch CE Relaxing the rule of ten events per variable in logistic and Cox regression Am J Epidemiol 2007 165 710 718 10.1093/aje/kwk052 17182981

